# GFF Utilities: GffRead and GffCompare

**DOI:** 10.12688/f1000research.23297.2

**Published:** 2020-09-09

**Authors:** Geo Pertea, Mihaela Pertea

**Affiliations:** 1 Center for Computational Biology, Johns Hopkins University, Baltimore, MD, 21218, USA; 2 Department of Biomedical Engineering, Johns Hopkins University, Baltimore, MD, 21218, USA

**Keywords:** gene annotation, transcriptome analysis, GTF and GFF file formats

## Abstract

**Summary:** GTF (Gene Transfer Format) and GFF (General Feature Format) are popular file formats used by bioinformatics programs to represent and exchange information about various genomic features, such as gene and transcript locations and structure. GffRead and GffCompare are open source programs that provide extensive and efficient solutions to manipulate files in a GTF or GFF format. While GffRead can convert, sort, filter, transform, or cluster genomic features, GffCompare can be used to compare and merge different gene annotations.

**Availability and implementation:** GFF utilities are implemented in C++ for Linux and OS X and released as open source under an MIT license  ( https://github.com/gpertea/gffread, https://github.com/gpertea/gffcompare).

## Introduction

Many biomedical research applications employ pipelines to systematically analyze the gene content in a genome. Due to the explosion in transcriptomic data available, these pipelines routinely involve processing enormous amounts of data, and therefore require efficient bioinformatics tools that can handle multiple annotation and sequence files in order to speed up the genomic analysis. Such tools usually exchange and employ information about genes, transcripts or other genomic features in a tab-delimited text file format commonly known as GFF (General Feature Format). This format describes the exact coordinates and attributes of genes, transcripts, and other features such as start and stop codons, coding sequences etc. As such, a typical line in the GFF format specifies a given feature by using the following fields: 


<seqname> <source> <feature> <start> <end> <score> <strand> <frame> <attributes>


where <seqname> provides the sequence name of the feature’s location, <source> is the program that generated that feature, <feature> gives the actual type of the feature, <start> and <end> are the start and end coordinates of the feature on the sequence, <score> is a floating-point number that represents the score attributed to that feature, <strand> gives the strand of the feature on the sequence, <frame> is used for a coding feature to indicate where the next codon begins relative to the 5' end, and <attributes> specify additional characteristics for the feature that depend on the specific version of the GFF format used and usually include at least a unique identifier for that feature.

GFF has many versions, including its latest version GFF3 ^1^ and the older GTF (Gene Transfer Format), sometimes also referred to as GTF2 ^2^. While the older GTF format is limited to the representation of gene and transcript locations and their structures, the newer GFF3 format can represent many more genomic features and annotations in a hierarchical fashion. Some transcript data or genome annotation is available from the source in only one of these formats, but an application may require the other format as input. The GffRead and GffCompare utilities can automatically recognize and work with both these file formats seamlessly, extract and select transcript features from data rich GFF3 annotation files, perform conversions from one format to another, and even convert files from and to other formats such as BED ^3^ or FASTA ^4^.

Annotation data from different sources may use different naming conventions for chromosomes and contigs, and GffRead can help with mapping such genomic sequence names and thus converting annotation from one reference naming convention to another. Gene prediction programs and transcript (RNA-Seq) assembly programs usually output their results in GTF or GFF3 format, and in such cases there is often a need to assess the accuracy of the predicted/assembled transcripts. GffCompare is designed to systematically compare one or more sets of transcript predictions to a reference annotation at different levels of granularity (base level, exon level, transcript level etc.), and in the process to provide a way to "annotate" such transcript predictions based on their overlaps or proximity to reference annotation transcripts. When multiple transcript files (samples) are provided, GffCompare generates a non-redundant combined set of transcripts, tracking structurally equivalent transcripts across multiple samples and classifying them according to their relationship to reference transcripts.

Due to their efficiency and user-friendly nature, both GffRead and GffCompare have already been used in many bioinformatics projects as integral parts of pipelines for genome annotation ^5– 7^, novel gene discoveries and characterizations ^8– 18^, gene structure reconstruction accuracy ^19– 21^, and gene annotation comparisons ^22– 25^ among others. In this paper we provide detailed descriptions of the specific functions provided by our GFF utilities.

## Methods

### Implementation

Both our utilities share a code base built around a C++ class called GffObj that implements many of the common GFF parsing and indexing functions. Because the GFF format has no requirements for grouping and sorting of hierarchically linked genomic features (e.g. a transcript feature can have one of its exons at the beginning of the file and another at the end of the file), the parser has to keep transcript data in memory until the whole file is parsed. Feature identifiers (like transcript IDs) are kept in string hashes for fast identification of hierarchical relationship between features. Reference sequence names and GFF attribute names are also stored in global string hashes with numeric IDs associated, while pointers to the genomic feature objects (GffObj) are stored in dynamic arrays sorted by the genomic location such that a binary search can be used for quick overlap verification. The code shared by these utilities also implements functions to test and classify the structural similarities and overlaps between transcripts in the same location on the genome.

***GffRead***. We initially implemented the GffRead utility as a fast tool for verification, filtering and conversion of the most popular annotation file formats, GTF and GFF3, and for quick extraction of transcript sequences from the genome sequence. With its many features added over time, GffRead is now a complex and versatile tool that can sort, filter, remap and even cluster transcripts into loci (based on exon overlaps) while optionally discarding "redundant" transcripts from an input GFF data. Different examples for the command lines used to perform all these functions are offered in the Use Cases section below.

GffRead parses the input records given in GTF, GFF3 or BED format, and stores them into an internal collection of GffObj data structures that can be easily sorted and filtered according to different criteria. For instance, GffRead can output only the subset of the input transcripts that are multi-exonic, or do not belong to pseudogenes (see Table 1 for a complete set of filtering options). Besides conversions between different GFF formats, GffRead has many additional output options (see Table 2). Among these is a user-defined tab-delimited format, with a line for each transcript and the columns defined by a custom list of some of the GFF columns and attributes in the input annotation file. If a genome sequence is provided, GffRead can also generate multiple additional sequence data files in FASTA format such as: (1) a file with the transcript sequences produced by extracting and concatenating all of the exon sequences of each transcript; (2) a file with all the protein-coding sequences in each transcript; or (3) a file with the amino-acid translations of the coding sequence of each transcript. If a FASTA index file (such as the one created by the samtools utility ^26^) is not present in the same directory with the genomic sequence, GffRead will first create one in order to accelerate the retrieval of the specific transcript sequences. If the transcripts in the annotation file have coding sequences (represented as CDS features in the file), GffRead can check their validity and add specific annotations to the output file, indicating if either the START or the STOP codons are missing in these transcripts or if there are in-frame STOP codons.

**Table 1.** GffRead options controlling the filtering of the input GFF3 data (transcripts).

-i *<maxintron>*	discard transcripts having an intron larger than <maxintron>
-l *<minlen>*	discard transcripts shorter than <minlen> bases
-r *<chr>*: *<start>*- *<end>[<strand>]*	only show transcripts overlapping coordinate range <start>..<end> on reference sequence <chr> (on strand <strand> if provided)
-R	for -r option discard all transcripts that are not fully contained within the given range
-U	discard single-exon transcripts
-C	coding only: discard transcripts that do not have CDS features
--nc	non-coding only: discard transcripts that have CDS features
-V	discard any coding transcripts having in-frame stop codons (requires -g)
-N	discard multi-exon mRNAs that have any intron with a non-canonical splice site consensus (i.e. not GT-AG, GC-AG or AT-AC)
-J	discard any transcripts that either lack initial START codon or the terminal STOP codon, or have an in-frame stop codon (i.e. only print mRNAs with a complete, valid CDS)
--no-pseudo	discard genes and their transcripts having features or attributes indicating a 'pseudogene'
-M/--merge	cluster the input transcripts into loci, discarding "duplicated" transcripts (those with the same exact introns and fully contained or equal boundaries)
-K	for -M option: also discard as redundant the shorter, fully contained transcripts (intron chains matching a part of the container)
-Q	for -M option, no longer require boundary containment when assessing redundancy (can be combined with -K); only introns have to match for multi-exon transcripts, and >=80% overlap for single- exon transcripts

**Table 2.** GffRead output options; default output consists of transcripts only, shown as GFF records with only the basic attributes kept (ID, Parent, geneID and gene_name if found).

-F	preserve all original GFF attributes (for non-exon features); repetitive/redundant exon/CDS attributes are merged into the parent transcript attributes
--keep-exon-attrs	for -F option, do not attempt to reduce redundant exon/CDS attributes
--keep-genes	in transcript-only mode (default), also preserve gene records
-P	add transcript level GFF attributes about the coding status of each transcript, including partialness or in-frame stop codons (requires -g)
--force-exons	make sure that output transcripts have "exon" features generated when they were not explicitly given in the input (e.g. CDS-only transcripts)
--gene2exon	for single-line genes not parenting any transcripts, add an exon feature spanning the entire gene (treat it as a transcript)
-Z	merge very close exons into a single exon (when intron size<4)
-w	write a FASTA file with spliced exons for each transcript
-x	write a FASTA file with spliced CDS for each GFF transcript
-y	write a protein FASTA file with the translation of CDS for each record
-T	main output is GTF instead of GFF3
--bed	main output is in BED format instead of GFF3
--table	output a simple tab delimited format instead of GFF, with columns having the values of GFF attributes given in <attrlist>; special pseudo-attributes (prefixed by @) are recognized: @id, @geneid, @chr, @start, @end, @strand, @numexons, @exons,@cds, @covlen, @cdslen If any of -w/-y/-x output files are enabled, the same fields (excluding @id) are appended to the definition line of corresponding FASTA records
*Output sorting options (by default the output is sorted by feature coordinates per reference sequence, with reference* *sequences shown in the order they were first encountered in the input):*	
--sort-alpha	reference sequences are sorted alphabetically
--sort-by <refseq.lst>	sort the reference sequences by the order their names are given in the <refseq.lst> file

The transcript clustering functions of GffRead can group each set of input transcripts into a locus, where all transcripts in a locus are on the same strand, and any two transcripts in that locus have at least one exonic interval overlap. When clustering is enabled, the GFF output will have a new 'locus' feature for each cluster with attributes listing all the transcript IDs (and gene IDs, if available) that belong to that cluster. Optionally, GffRead can identify transcripts that are structurally "matching" or "equivalent", defined as transcripts that share all their introns, or have more than 80% of their length overlap in the case of single exon transcripts. GffRead can also discard redundant transcripts (either matching or contained within other transcripts) from the output, providing the user with the ability to choose among merging strategies with different levels of stringency when assessing redundancy in such cases.

***GffCompare***. GffCompare is a generic, standalone tool for merging and tracking transcript structures across multiple samples and comparing them to a reference annotation. Initially written based on the CuffCompare utility program included with the Cufflinks suite ^27^, GffCompare has the following main functions:

1) merge structurally equivalent transcripts and transcript fragments (transfrags) across multiple samples;
2) assess the accuracy of the assembled transcripts from an RNA-Seq sample by comparing it to known annotation; and
3) track, annotate, and report all structurally distinct transfrags across multiple samples.

**Figure 1.**
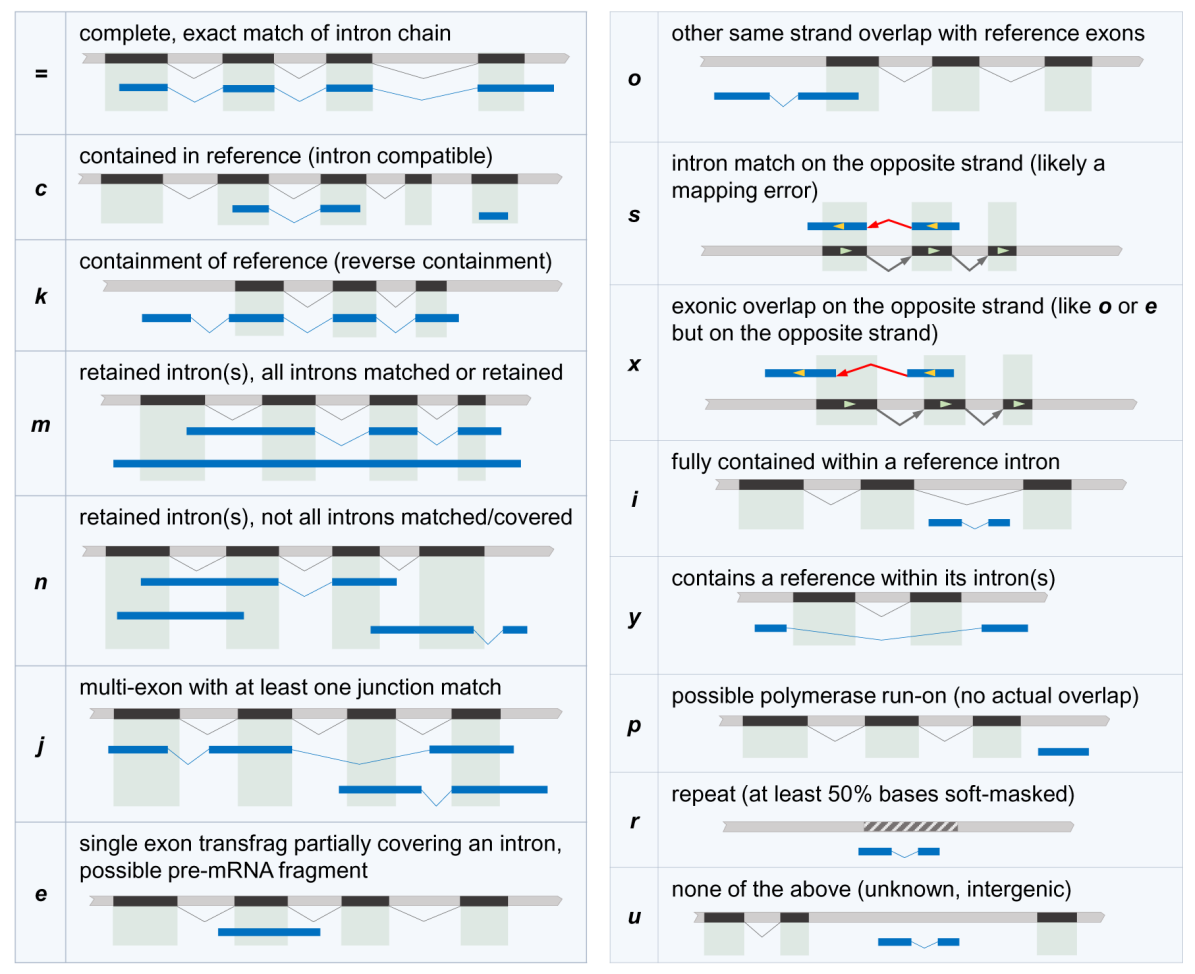
Transcript classification codes based on their relationship to reference transcripts, as generated by GffCompare. Reference exons and transcripts are shown in black, transcripts to be classified are shown in blue, and hashed regions represent repeated regions in the genome. For example, the transcript in blue on the uppermost left panel is labeled “=” because all of its introns precisely match the annotation in black.

The last two purposes require the user to provide a known reference annotation file that GffCompare then uses to classify all the transcripts in the input samples according to the reference transcript that they most closely overlap ( Figure 1). To assess the accuracy of transcriptome assemblies, GffCompare reports several accuracy metrics previously employed for gene prediction evaluation ^28^. These metrics include sensitivity and precision as well as the number of novel or missed features, and the metrics are computed at various levels (base, exon, intron chain, transcript, or locus). More details about how to obtain the different reports provided by GffCompare can be found in the Use Cases section.

Some pipelines can produce a very large number of transcripts that need to be evaluated; e.g. when merging the transcript assemblies from tens or hundreds of RNA-Seq experiments. Because GffCompare always loads the entire transcript data into memory for clustering, running GffCompare on such large GTF/GFF files could be slow and memory intensive. One may be interested only in how these transcripts overlap the reference annotation, and then only wish to further analyze those transcripts that have specific types of overlaps with the reference annotation transcripts. GffCompare also only produces the best match of a transcript to a reference annotation, but for each transcript we might want to know all possible reference matches. In order to address these needs, we built TrMap ("Transcript vs. reference Mapping"), a program that we distribute along with GffCompare and that was designed to avoid using a large amount of memory by streaming the input transcript data. TrMap first loads the reference annotation into an interval tree data structure ^29^, and then for each query transcript it reports all the reference transcripts that overlap it, along with their overlap classification codes. These are the same classification codes described in Figure 1, with the exception of codes *p*, *r*, and *u* which are reserved for transcripts that do not overlap reference transcripts and represent transcripts that are single exon and nearby genes ( *p*), repeats outside of genes ( *r*), and intergenic ( *u*).

### Operation

This software can be built on a Linux or MacOS system with no other library dependencies. A GNU C++ compiler (g++) is required for compilation (on Linux at least g++ version 4.5 is required). The release packages on Github include precompiled binaries for Linux and MacOS that can be used directly instead of having to build the programs from source. Linux compatibility goes back as far as RedHat Enterprise Linux 5, while on MacOS the programs can run on systems as old as OS X 10.7 (Lion). We also provide the gffread, gffcompare and trmap executables. These are supposed to be used as command line programs, in a Linux/Unix shell, in a terminal or a script. All programs take GFF3, GTF or BED files as their (main) input files. Both packages require the shared code provided in GCLib ( https://github.com/gpertea/gclib ^30^).

## Use cases

The following sections illustrate different use cases for our utilities. All the files used in the examples below as well as their output are included in the gffread and gffcompare Github release packages ( https://github.com/gpertea/gffread ^31^, https://github.com/gpertea/gffcompare ^32^) so that the interested user can try these examples for themselves.

### Basic usage examples of the GffRead utility

The program GffRead can be used to validate, filter, convert and perform various other operations on GFF files (see Table 1 and Table 2 for the full list of usage options). For instance, GffRead can be used to simply read an annotation file in a GFF format, and print it in either GFF3 (default) or GTF2 format (with the -T option), while optionally discarding any non-essential attributes, and fixing some potential issues with the input file. The command line for such a quick cleanup and a quick visual inspection of a given GFF file would be:


gffread -E annotation.gff -o ann_simple.gff


This will show the minimalist GFF3 re-formatting of the transcript records found in the input file ( annotation.gff in this example) which could be given in either GFF3 or GTF2 format. The - E option directs GffRead to "expose" (display warnings about) any potential issues encountered while parsing the input file.

In order to obtain the GTF2 version of the same transcripts, the - T option should be added:


gffread annotation.gff -T -o annotation.gtf


GffRead can be used to generate a FASTA file with the DNA sequences for all transcripts in a GFF file. For this operation a FASTA file with the genomic sequences have to be provided as well. This can be accomplished with a command line like this:


gffread -w transcripts.fa -g genome.fa annotation.gff


The file genome.fa in this example would be a multi-FASTA file with the chromosome/contig sequences of the target genome. This also requires that every contig or chromosome name found in the 1st column of the input GFF file ( annotation.gtf in this example) must have a corresponding sequence entry in the genome.fa file.

### Basic usage example of the GffCompare utility

The program GffCompare can be used to compare, merge, annotate and estimate accuracy of one or more GTF/GFF files (the “query” files), when compared with a reference annotation (also provided as GTF/GFF). A basic command line to compare a list of GTF files to a reference annotation file is:


gffcompare -r annotation.gff transcripts.gtf


The reference annotation is specified in the annotation.gff file and transcripts.gtf represents the query file (more than one query file can be provided). Unless the -o option was provided, the output will be found in multiple files with the prefix “gffcmp.”. A list of the more important options for the GffCompare utility is provided in Table 3.

**Table 3.** GffCompare options.

-i <input_gtf_list>	provide a text file with a list of (query) GTF/GFF files to process instead of expecting them as command line arguments (useful when a large number of GTF files should be processed)
-r <reference.gff>	provides reference annotation file (GTF/GFF)
-R	for -r option, consider only the reference transcripts that overlap any of the input transfrags (Sensitivity correction)
-Q	for -r option, consider only the input transcripts that overlap any of the reference transcripts (Precision correction); this will discard all novel loci
-M	discard (ignore) single-exon transfrags and reference transcripts
-N	discard (ignore) single-exon reference transcripts
-D	discard "duplicate" query transfrags (i.e. those with the same intron chain) within a single sample
-S	like -D, but stricter duplicate checking: only discard matching query or reference transcripts (same intron chain) if their boundaries are fully contained within other, larger or identical transfrags
--no-merge	disable close-exon merging (default: merge exons separated by "introns" shorter than 5 bases)
-s <genome_file>	path to genome sequences (optional); this can be either a multi-FASTA file or a directory containing single-FASTA files (one for each contig); repeats must be soft-masked (lower case) in order to be able to classify transfrags as repeats
-T	do not generate .tmap and .refmap files for each input file
-e	max. distance (range) allowed from free ends of terminal exons of reference transcripts when assessing exon accuracy (default: 100)
-d	max. distance (range) for grouping transcript start sites (default: 100)
-V	verbose processing mode (also shows GFF parser warnings)
--chr-stats	the .stats file will show summary and accuracy data for each reference contig/ chromosome separately
-p <cprefix>	the name prefix to use for consensus transcripts in the <outprefix>.combined. gtf file (default: 'TCONS')
--debug	enables -V and generates additional files: <outprefix>.Q_discarded.lst, <outprefix>.missed_introns.gff, <outprefix>.R_missed.lst
Options for the combined GTF output file:	
-o <outprefix>	provides a prefix for all output files
-C	discard matching and "contained" transfrags in the GTF output (i.e. collapse intron- redundant transfrags across all query files)
-A	like -C but does not discard intron-redundant transfrags if they start with a different 5' exon (keep alternate TSS)
-X	like -C but also discard contained transfrags if transfrag ends stick out within the container's introns
-K	for -C/-A/-X, do NOT discard any redundant transfrag matching a reference

### Transcript accuracy estimation with GffCompare

GffCompare can be used to assess the accuracy of transcriptome assemblies produced by programs like StringTie ^19^ with respect to a known reference annotation. To this end, GffCompare reports various statistics related to the accuracy of the input transcripts compared to the reference annotation in the <outprefix>.stats file. Among these statistics are sensitivity and precision values computed at various levels (base, exon, intron chain, transcript, locus), which are calculated as:

$$\begin{array}{l} Sensitivity\;=\;TP/(TP+FN)\\ Precision\;=\;TP/(TP+FP) \end{array}$$

where *TP* stands for "true positives", or query features (bases, exons, introns, transcripts, etc.) that agree with the corresponding reference annotation features; *FN* means "false negatives", i.e. features that are found in the reference annotation but are not present in the input data; *FP* (“false positives”) are features present in the input data but not confirmed by any reference annotation data. Notice that *FP*+ *TP* amounts to the whole input set of query features in the input file. If multiple query GTF/GFF files are given as input, these metrics are computed separately for each sample.

*Sensitivity* and *Precision* values are estimated at various levels, which are largely an increasingly stringent way of evaluating the accuracy/correctness of a set of predicted transcripts (transfrags), when compared to the reference annotation provided with the - r option. The six different levels that GffCompare uses are described below: 

1) *Base level.* At the base level, *TP* represents the number of exon bases that are reported at the same coordinate on both the query transcripts and any reference transcript, *FN* is the number of bases in reference data exons that are not covered at all by any of the query exons, and FP is the number of bases which are covered by predicted transcripts' exons but not covered by any reference transcript exons.

2) *Exon level.* We define the *TP*, *FN*, and FP values at the exon level similar to the base level, but now the unit of comparison is the exon interval on the genome, i.e. if an exon of the predicted transcript overlaps and matches the boundaries of a reference transcript exon, then it is counted as a *TP*.

3) *Intron Level.* Intron intervals are the units that are matched at the intron level, therefore each intron of the predicted transcript is checked against any introns of the reference transcripts in the same region and if there is one with the same exact start-end coordinates, it is counted as a *TP*.

4) *Intron chain level.* At this level we count as a *TP* any query transcript for which all of its introns can be found, with the same exact intron coordinates as in a reference transcript that has the same number of introns. Matching all the introns at this level implies that all the internal exons also match, but this might not be true for the external boundaries of the terminal exons.

5) *Transcript level.* Note that intron chain level values are calculated only by looking at multi-exon transcripts, so it completely ignores the single-exon transcripts, which can be quite numerous in a RNA-Seq experiment (possibly due to a lot of transcriptional and alignment noise). The transcript level considers single-exons as well. A *TP* at this level is defined as a full exon chain match between the predicted transcript and a reference transcript, where all internal exons match and the outer boundaries of the terminal query exons can only slightly differ from the reference exons (with at most 100 bases by default). Also GffCompare considers single-exon transcripts as matching an overlapping single-exon reference transcript if there is a significant overlap between the two (more than 80% of the longer transcript by default).

6) *Locus level.* At this level GffCompare considers that an observed locus, defined as a cluster of exon-overlapping transcripts, matches a similarly built reference locus if at least one predicted transcript has a transcript level match with a reference transcript in the corresponding reference locus.

Other statistics reported by GffCompare are the number of missed or novel exons, missed or novel introns and missed or novel loci. Note that in order to properly evaluate precision and sensitivity when comparing two sets of transcripts, special care must be taken for duplicated (or redundant) entries within each set. GffCompare uses different levels of stringency of what to consider duplicated transcripts, depending on the option given in its input (see options - D, - S, - C, - A, - X in Table 3).

### Merging structurally equivalent transcripts with GffCompare

When multiple input GTF/GFF files are provided, GffCompare reports a GTF file named <outprefix>.combined.gtf containing the union of all transfrags in each sample. If a transfrag with the same exact intron chain is present in both samples, it is thus reported only once in the output file.

*The "super-locus" concept*

A super-locus is a region of the genome where predicted transcripts and reference transcripts get clustered together by exon overlaps. When multiple GFF files are provided as input to GffCompare, this clustering is performed across all the input files. Due to the transitive nature of this clustering, these super-loci can occasionally get very large, sometimes merging a few distinct reference gene regions together, especially if there is a lot of transcription or alignment noise around the individual gene regions. For each super-locus, GffCompare assigns a unique identifier with the *XLOC_* prefix.

### Annotating transcripts with GffCompare

One can run GffCompare on a single GTF/GFF input file using with the - r option (which provides a reference annotation), and without any specific options to remove redundant transfrags (such as the - D, - S, - C, - A, - X options) to produce a GTF file called <outprefix>.annotated.gtf that contains all the input transcripts annotated with several additional attributes: xloc, tss_id, cmp_ref, and class_code. The xloc attribute specifies the super-locus a specific transcript belongs to. The tss_id attribute uniquely identifies the transcription start for that transcipt, and using this value the user can quickly see which transcripts use the same transcription start, or how many different transcription starts are present in a locus. The cmp_ref gives the closest reference transcript (where applicable), while the relationship to this reference transcript is given by the class_code attribute. The possible values for the class_code attribute are listed in Table 4.

**Table 4.** Transcript classification codes (listed in decreasing order of priority).

Code	Relationship to reference transcript
=	complete, exact intron chain match
c	contained in reference transcript (intron compatible)
k	contains reference transcript (reverse containment)
m	retained intron(s) compared to reference, full intron chain match everywhere else
n	completely overlaps intron from reference transcript, partial or no intron chain match everywhere else
j	multi-exon with at least one junction match
e	single exon that partially covers an intron from reference
o	other same strand overlap with reference exons
s	intron match on the opposite strand (likely a mapping error)
x	exonic overlap on the opposite strand
i	fully contained within a reference intron
y	contains a reference within its intron(s)
p	possible polymerase run-on (close to reference but no overlap)
r	repeat (at least 50% bases are soft-masked)
u	none of the above (unknown, intergenic)

### Tracking transcripts with GffCompare

GffCompare can also be used to track all transcripts that are structurally equivalent among the different input files. GffCompare considers transcripts matching (or structurally equivalent) if all their introns are identical. Note that matching transcripts are allowed to differ on the length of the first and last exons, since these lengths can usually vary across samples for the same biological transcript. A list of all matching transcripts is reported in a file called <outprefix>.tracking in which each row represents a transcript. The first column in this file represents a unique id assigned to that transcripts. The second file represents the super-locus that contains that transcript. If GffCompare was run with the - r option, the 3 ^rd^ and 4 ^th^ columns contain the reference annotation transcript that was found to be closest to the transcript and the classification code (as specified by Table 4) that specifies the relationship between these two transcripts, respectively. The rest of the columns show the corresponding transcript from each input file in order. An example and a brief description for each column are given in Table 5.

**Table 5.** Description of the columns in the <outprefix>.tracking generated by GffCompare when run on *N*≥1 input files.

Column number	Column name	Example	Description
1	Query transfrag id	TCONS_00403479	A unique internal id for the transfrag
2	Query locus id	XLOC_006534	A unique internal id for the super-locus containing these transcripts across all samples and the reference annotation
3	Reference gene id and transcript id	TCEA3|rna-XM_006710864.2	The gene name and transcript ID of the reference record associated to this transcript (separated by '|'), or ‘-’ if no such reference transcript is available
4	Class code	j	The type of overlap or relationship between the reference transcripts and the transcript structure represented by this row
5.. *N*	Corresponding transcript in input file *n*	q1:STRG.377|STRG.377.2|10| 0.304785|0.760185|2.205239 |2767	q *n*: *<gene_id>*| *<transcript_id>*| *<num_exons>* | *<FPKM>*| *<TPM>*| *<cov>*| *<len>*

In order to quickly see which reference transcripts match which transcripts from a sample file, two other files, called <outprefix>. <input_file>. refmap and <outprefix>. <input_file>. tmap are also created for each query <input_file>. The <outprefix>. <input_file>. refmap file is a tab-delimited file that has a row for each reference transcript that either fully or partially matches a transcript from the given input file. Its columns are described in Table 6. Conversely, the <outprefix>. <input_file>. tmap file has a row for each input transcript, while the columns in this file (as detailed in Table 7) describe the most closely matching reference transcript for that transcript.

**Table 6.** Description of the columns in the <outprefix>.<input_file>.refmap file.

Column number	Column name	Example	Description
1	Reference gene name	Myog	The gene_name attribute of the reference GTF record for this transcript, if present. Otherwise gene_id is used.
2	Reference transcript id	uc007crl.1	The transcript_id attribute of the reference GTF record for this transcript.
3	Class code	c	The type of match between the query transcripts in column 4 and the reference transcript. One of either ‘c’ for partial match, or ‘=’ for full match.
4	Matches	STRG.223|STRG.223.1,STRG.224| STRG.224.1	A comma separated list of transcripts matching the reference transcript.

**Table 7.** Description of the columns in the <outprefix>.<input_file>.tmap file.

Column number	Column name	Example	Description
1	Reference gene name	Myog	The gene_name attribute of the reference GTF record for this transcript, if present. Otherwise gene_id is used.
2	Reference transcript id	uc007crl.1	The transcript_id attribute of the reference GTF record for this transcript
3	Class code	c	The type of relationship between the query transcripts in column 4 and the reference transcript (as described in the Class Codes section below)
4	Query gene id	STRG.23567	The query (e.g., Stringtie) internal gene id
5	Query transcript id	STRG.23567.0	The query internal transcript id
6	Number of exons	7	The number of exons in the query transcript
7	FPKM	1.4567	The expression of this transcript expressed in FPKM
8	TPM	0.000000	the estimated TPM for the transcript, if found in the query input file
9	Coverage	3.2687	The estimated average depth of read coverage across the transcript.
10	Length	1426	The length of the transcript
11	Major isoform ID	STRG.23567.0	The query ID of the gene's major isoform
12	Reference match length	4370	The length of the longest overlap with a reference, ‘-’ if there is no such exonic overlap

### Overlap classification for a large set of transcripts with TrMap

The utility TrMap was designed for large scale overlap analysis of streaming transcript prediction data (millions of transcripts) with a reference annotation data set. Particularly, TrMap performs detection and classification of all the overlaps found between the streamed transcripts and the reference annotation transcripts.

The program *trmap* is distributed with GffCompare and a basic usage for it is shown below:


trmap [-S] [-o ] <ref_gff> <query_gff>
Positional arguments:
  <ref_gff>    reference annotation file name (GFF/BED format)
  <query_gff>  query file name (GFF/BED format) or "-" for stdin
Options:
  -o <outfile> write output to <outfile> instead of stdout
  -S           report only simple reference overlap percentages, without
               classification (one line per query)


The default output is a pseudo-FASTA format showing a record for each query transcript that had at least one reference overlap. The query transcript is shown in the header of the record, with space delimited fields showing the genomic location and strand. Each reference overlap follows, as a line with tab delimited fields, starting with the "classification code" for the overlap and then providing the genomic location of the transcript (chromosome, strand, transcript-start, transcript-end, reference_transcriptID, exons).

The exons for both query and reference transcripts are shown as comma delimited lists of intervals. These are all 1-based coordinates like in the GTF/GFF format (even when input is BED).

## Conclusions

GffRead and GffCompare provide comprehensive features for converting, filtering, manipulating, clustering, combining and classifying transcript data from GFF files. Due to their ability to process hundreds or even thousands of transcript files at the same time, they can be used for large scale genome data analysis by many bioinformatics analysis pipelines.

## Data availability

### Underlying data

*All data underlying the results are available as part of the article and no additional source data are required.*

## Software availability

The source packages for the latest release, with precompiled binaries and online manuals, are available at http://ccb.jhu.edu/software/stringtie/gff.shtml.

Source code available from: https://github.com/gpertea/gffread

Archived source code at time of publication: http://doi.org/10.5281/zenodo.3755686 ^31^

License: MIT

Source code available from: https://github.com/gpertea/gffcompare

Archived source code at time of publication: http://doi.org/10.5281/zenodo.3755715 ^32^

License: MIT

Source code available from: https://github.com/gpertea/gclib

Archived source code at time of publication: http://doi.org/10.5281/zenodo.3758741 ^30^

License: Artistic License 2.0
