# Supplementary material for: GFF Utilities: GffRead and GffCompare
Source: F1000Res. 2020 Sep 9;9:ISCB Comm J-304. Originally published 2020 Apr 28. [Version 2] doi: 10.12688/f1000research.23297.2 (PMC7222033; doi:10.12688/f1000research.23297.2)
Supplement: Peer review history

# Peer review: Reviewer response for version 1

**DOI:** 10.5256/f1000research.25718.r62867

**Published:** 2020-06-02

**Authors:** Rob Patro

**Affiliations:** 1 Department of Computer Science, University of Maryland, College Park, MD, USA

In this manuscript, the authors describe the GFF Utilities suite, which is comprised of the GffRead, GffCompare, and trmap utilities. These tools long predate the article and have been in relatively widespread use for some time. For example, GffRead is commonly used to convert files to proper GFF3 format (and to record or catalog formatting errors), as well as to extract the sequences of transcripts (or related features) from a GFF file and associated genome. GffCompare is commonly used as a standard tool in the evaluation of genome-guided assembly, to compare predicted transcripts against an annotation, as well as to merge transcriptome assemblies across multiple samples while discarding duplicates. It is valuable, I think, to have an article describing these tools that can also be cited as they are used and built upon in the community. I found the visual description of the different transcript classification codes (Figure 1) particularly useful. I was able to download and build the source easily according to the instructions in the respective README files. In addition to the source and pre-compiled builds, it is worth noting that both GffRead and GffCompare are available through Bioconda, though I am not sure if the authors of the current manuscript are the Bioconda maintainers for these packages.

   Overall, the article is clear and describes the tools well. I have only very minor comments.

  

-  pg 6: "produced by programs like StringTie in respect to" -> "produced by programs like StringTie with respect to"
-  I tried compiling both GffRead and GffCompare under multiple versions of G++. With G++5.5.0, everything worked well with only two warnings (a strict overflow warning in gfoAdd() and an unused variable warning for isCodonTableReady). However, when I compiled with G++9.2.0, there were multiple warnings in the GVec class about potentially incorrect uses of memset, which should actually not be an issue as the uses are present in a branch that first checks that the type being set is "primitive".  Peeking into the Makefile, I noticed that a particular flag is set to disable this warning under newer versions of G++ (presumably, it does not occur in earlier versions). However, in the line of the makefile that checks this, `g++` is hard-coded, rather than making use of the potentially modified `${CXX}` variable. Changing `g++` to `${CXX}` in the lines of the makefiles defining the `GCCV8` variable resolved this behavior.
    
- Finally, this is not an issue with the current manuscript, but rather a general note. In addition to powering the GffRead and GffCompare tools, the Gff utilities source code provides a powerful programmatic interface (API) to parse, manipulate and process GFF and GTF files in the C++ language. If the authors feel it appropriate, a similar note or article describing the features and capabilities of that library may be of interest to a related (though admittedly smaller) part of the community.

Are the conclusions about the tool and its performance adequately supported by the findings presented in the article?

Yes

Is the rationale for developing the new software tool clearly explained?

Yes

Is the description of the software tool technically sound?

Yes

Are sufficient details of the code, methods and analysis (if applicable) provided to allow replication of the software development and its use by others?

Yes

Is sufficient information provided to allow interpretation of the expected output datasets and any results generated using the tool?

Yes

Reviewer Expertise:

NA

I confirm that I have read this submission and believe that I have an appropriate level of expertise to confirm that it is of an acceptable scientific standard.

# Peer review: Reviewer response for version 1

**DOI:** 10.5256/f1000research.25718.r62863

**Published:** 2020-05-13

**Authors:** Michael I. Love

**Affiliations:** 1 Department of Biostatistics, University of North Carolina-Chapel Hill, Chapel Hill, NC, USA

This software tool article motivates and describes the use of GffRead and GffCompare, two utility programs for manipulating GFF and GTF files, including a comparison of one set of transcripts to a reference. The article is well written and clearly describes the use cases for the software. I have downloaded the two programs onto a laptop running OS X and have used the precompiled Mac binaries as well as building the tools from source. I ran GffRead to change a Gencode GTF file to GFF, and to extract spliced transcript sequences from a genome FASTA. I ran GffCompare to compare two Gencode releases of a GTF file. The programs were easy to run, took at most a few minutes on a laptop (for comparison), and are both well documented. I have only minor comments (spelling/formatting):

- RNA-Seq is used twice, while RNA-seq is used once.
- Table 1 has "(strand )" with a space (angle brackets not allowed in the report, so I use parens).
- Table 2 row two, column two has a line break.
- Transfrags are defined in this article but not in the online documentation.
- Under "Use Cases", there is a "fasta" not capitalized.
- Under "Transcript accuracy estimation with GffCompare", perhaps "with respect to a known reference annotation".
- Under "Annotating transcripts with GffCompare", should be "this reference transcript".
- Under "Tracking transcripts with GffCompare", "(input_file)" is italicized.

Are the conclusions about the tool and its performance adequately supported by the findings presented in the article?

Yes

Is the rationale for developing the new software tool clearly explained?

Yes

Is the description of the software tool technically sound?

Yes

Are sufficient details of the code, methods and analysis (if applicable) provided to allow replication of the software development and its use by others?

Yes

Is sufficient information provided to allow interpretation of the expected output datasets and any results generated using the tool?

Yes

Reviewer Expertise:

RNA-seq bioinformatics, biostatistics, open source scientific software development

I confirm that I have read this submission and believe that I have an appropriate level of expertise to confirm that it is of an acceptable scientific standard.

# Peer review: Reviewer response for version 1

**DOI:** 10.5256/f1000research.25718.r62865

**Published:** 2020-05-12

**Authors:** Andreas Stroehlein

**Affiliations:** 1 Department of Veterinary Biosciences, Melbourne Veterinary School, Faculty of Veterinary and Agricultural Sciences, The University of Melbourne, Parkville, Vic, Australia

The authors provide a concise and useful summary of two widely-used tools for genomic analysis, GffRead and GffCompare. The paper is written well and provides clear guidelines and examples on how to use those tools. I only have very minor suggestions and overall have no objections to this work being approved for indexing:

  

- Ref. 1: This is a great reference that describes the GFF3 format in detail. Nevertheless, I think the present paper would benefit from a concise, technical summary of the format in the Introduction section and maybe a sentence about issues surrounding different definitions of the format and the availability of GFF3 validators.
- Page 2, first paragraph, “rich GFF3 annotation files”: Does this mean “data-rich”? Maybe reword to clarify. What does “rich” entail?
- The same paragraph, “perform conversions from one from to another”: I think this is meant to read” from one format to another”.
- Page 6, “Basic usage example of the GffCompare utility”
  This paragraph states that query input can be GFF or GTF (text says GFF and example uses a GTF file), but Table 3 states for the -i option “provide a text file with a list of (query) GTF files”. Could you please clarify whether the -i option also accepts GFF files (similar to the description for -r option)?
- Page 8, “The “super-locus” concept”, “When multiple GFF files with are provided as input to GffCompare”: This seems to be a typo, or there is a word missing in the sentence? Remove “with”?
- Page 8, “Annotating transcripts with GffCompare”, 3 ^rd^ line: Change “produce an GTF file called” to “produce a GTF file called”.

Are the conclusions about the tool and its performance adequately supported by the findings presented in the article?

Yes

Is the rationale for developing the new software tool clearly explained?

Yes

Is the description of the software tool technically sound?

Yes

Are sufficient details of the code, methods and analysis (if applicable) provided to allow replication of the software development and its use by others?

Yes

Is sufficient information provided to allow interpretation of the expected output datasets and any results generated using the tool?

Yes

Reviewer Expertise:

Genomics, Bioinformatics, Parasitology

I confirm that I have read this submission and believe that I have an appropriate level of expertise to confirm that it is of an acceptable scientific standard.
